# Mechanical and Microstructural Properties of HDPE Pipes Manufactured via Orbital Friction Stir Welding

**DOI:** 10.3390/ma15113810

**Published:** 2022-05-27

**Authors:** Hesam Mehdikhani, Amir Mostafapour, Hossein Laieghi, Reza Najjar, Francesca Lionetto

**Affiliations:** 1 Faculty of Mechanical Engineering, University of Tabriz, Tabriz P.O. Box 51666-16471, Iran; hesammeh75@gmail.com; 2 Department of Mechanical Engineering, Gazi University, Ankara 06570, Turkey; hossein.laieghi@gmail.com; 3 Additive Manufacturing Technologies Research and Application Center-EKTAM, Gazi University, Ankara 06560, Turkey; 4 Polymer Research Laboratory, Faculty of Chemistry, University of Tabriz, Tabriz P.O. Box 51666-16471, Iran; najjar@tabrizu.ac.ir; 5 Department of Engineering for Innovation, University of Salento, Via Monteroni, 73100 Lecce, Italy

**Keywords:** HDPE pipe, orbital friction stir welding (OFSW), tool design, morphology, mechanical properties, microstructural evaluation

## Abstract

In recent decades, extensive research has been performed on the friction stir welding of flat-shaped materials while pipe welding, particularly polymer pipes, still encounters challenging issues. This work presents a feasible route for joining high-density polyethylene (HDPE) pipes using an orbital friction stir welding (OFSW) set-up properly designed with a retractable pin tool. Fully consolidated joints were achieved using a portable heating-assisted OFSW system suited for on-site pipeline welding. The obtained joined pipes were characterized by a high-quality weld surface and a lack of defects arising from the tool-pin hole. The samples welded with the optimum parameters presented comparable properties with the base materials and even a slight increase in the tensile strength. The highest tensile and impact strengths were 14.4 MPa and 2.45 kJ/m^2^, respectively, which is 105% and 89% of those of the base material. XRD, FTIR, and SEM were also applied to assess the property changes in the HDPE pipes after the FSW process. The morphological analysis evidenced that the crystalline structure of the welded sample was similar to that of the base material, proving the effectiveness of the proposed technology.

## 1. Introduction

During recent decades, polymer materials have attracted industrial attention, benefiting from lower environmental impacts, costs, and weight [1]. Polymers are increasingly being applied in the pipeline industry thanks to their advantages, such as a long-term service life; high resistance to corrosion, abrasion, and chemicals; strong, durable, flexible, and lightweight; long-length pipe with leak-proof joints; low labor requirements for installations; and significant overall cost savings. Polyethylene is an ideal material for a broad variety of piping applications, such as potable water service or distribution lines, natural gas distribution, lawn sprinklers, sewers, waste disposal, and drainage lines. Due to the increasing applications in several fields and the need for fast production and installation, new and efficient welding methods are urgently required [2].

Joining methods such as welding are also required for creating complex and large structures for thermoplastic polymers, including polyethylene [3]. The welding methods for polymers can be classified into two principal groups according to the heat generation mechanism: methods comprising mechanical movement (spin, ultrasonic, and friction welding) and methods involving external heating (hot plate, hot gas, resistive, and implant welding). The joining of axisymmetric thermoplastic geometries has fewer welding options to obtain quality parts. Among the available methods, spin welding is particularly applied to this group of materials. In this method, two rotating parts’ surfaces are welded by rubbing them against each other under pressure. Almost all thermoplastic materials can be joined utilizing spin welding and there is no limitation on the welded parts’ size (over 1 m diameter). Bindal et al. [4] studied the impact of the axial pressure and rotation rate on the spin-welded joint overlap of a polypropylene pipe. Two injection molded circular parts were successfully spin welded in a shear joint configuration. The fast speed and reliability of the friction spin welding technique for circular weld geometries have been pointed out by authors.

Another method for joining circular parts is ultrasonic welding, employing high-frequency and low-amplitude vibration to achieve heat generation at the interface of the welded parts. The ultrasonic welding method has acquired prominence due to its higher strength and speed and its applicability to a wide variety of materials. Masuzawa et al. [5] found torsional ultrasonic welding was an effective way to join acrylic resin pipes through control of the process parameters. During the ultrasonic welding, the static pressure applied to the weld joint and the horn pressure significantly affected the welding time and joint appearance. Dell’Anna et al. [6] applied the in situ ultrasonic welding method to manufacture composite pipes from thermoplastic-based composite tapes. Unidirectional tapes of E-glass-reinforced amorphous poly(ethylene terephthalate) were laid up and consolidated in a filament winding machine modified with a set-up enabling ultrasonic welding. The proposed technique is also applicable in pipe joining and composite repairing. To the authors’ knowledge, and according to the mentioned research works, joining long and tall parts can be effectively welded through spin welding in comparison with ultrasonic welding, particularly if parts have openings. However, one of the main constraints of the spin welding technique is the non-uniform distribution of heat at the interface of joints. Thus, hollow parts possessing thin walls are far more appropriate for welding. Other drawbacks are the critical preparation of specimens and joint design limitations.

Friction stir welding (FSW), owing to its low process costs and time and higher joint quality and low temperature, has the potential to drastically reduce the preparation time, material waste, and common deficiencies associated with other joining techniques. Friction stir welding as a solid-state joining technique was invented and developed by the Welding Institute (TWI), UK, in 1991 [7]. During FSW, a non-consumable rotational tool is plunged into the interfaces of two sheets, generating frictional heat derived from the tool rotation rate to mix and join the welded materials [8,9]. This method, in the primary steps, was utilized to join aluminum alloy plates. However, in recent years, the FSW technique has been successfully employed to join polymers [10,11]. Due to the different chain lengths of polymer macromolecules, the FSW of polymers cannot be an absolutely solid-state process. During FSW, the shorter chains can experience their melting points while some longer chains might be in their solid state. With regard to the FSW of thermoplastic polymers, some research works have focused on the welding parameters, including the tool rotation rate, tool traverse speed, and tool design, to prevent defect formation and improve the weld strength. Bozkurt [12] analyzed the impact of the tool rotational speed, tool traverse speed, and tilt angle on the tensile strength of HDPE sheets. According to the results obtained by this author, the rotational speed contribution was prominent in the FSW of joints (73.85%), whereas the tilt angle had a slight effect (5.96%). Bilici et al. [13] probed the strength of FSSW HDPE plates in relation to different sets of process parameters. They identified optimal values of the tool rotation rate, dwell time, and tool plunge depth of 700 rpm, 60 s, and 6.2 mm, respectively. The weld strength of FSSW sheets that used these optimal process parameters was improved by about 40% compared to ones welded with the initial welding conditions. Huang et al. [1] employed an appropriate combination of the stationary shoulder and a taper pin with a screw thread and triple facets for friction stir lap welding between PEEK reinforced with 30% short carbon fiber and AA2060-T8 plates to achieve the desired joint integrity and mechanical properties. According to their obtained results, a rotation rate of 1600 rpm was identified as the optimum value to achieve a maximum shear strength of 18 MPa.

Another route for improving the mechanical and microstructural properties in the FSW process of polymers is the enhancement of the material properties with nanomaterials. This approach can promote the replacement of metallic components even in structural applications [14]. Laieghi et al. [15] used halloysite nanotubes as a reinforcement agent in the FSW of polyamide-6/nitrile butadiene rubber. They optimized the processing parameters, including the tool rotation rate, tool traverse speed, and plunge depth, with and without the heating-assisted tool system. Well-balanced mechanical properties of the heat-assisted FSW welded samples were obtained under proper selection of the welding and material parameters. Another example of an investigation of the material parameters is the use of dissimilar FSW of HDPE and ABS in the presence of multi-wall carbon nanotubes (MWCNTs), which was conducted by Gao et al. [16]. At the joint interface, MWCNTs led to an increment in the tensile strength and elongation, owing to an increase in the thermal conductivity. However, a considerable reduction in the hardness occurred due to the addition of MWCNTs to the joints. In complex assemblies (vehicle bodies), thermoplastic composites are usually joined to metallic parts. Friction-based joining of hybrid structures has previously been examined by Lambiase et al. [17]. A comprehensive overview of the available advanced joining processes, including friction-oriented ones, for metal-composite and metal-polymer hybrid structures is provided in this study.

Although prominent attempts have been applied to the FSW of various materials, most of the research has involved flat-shaped plates and the number of investigations focusing on the FSW of pipes is very limited. This is because pipe joining via FSW is still facing difficulties such as the design of a unique mechanism and tooling system. For the successful joining of pure Cu and Al3003 pipes with a 19-mm outer diameter, Chen et al. [18] designed a special FSW system applicable to air-conditioning and heating systems. Welding temperature changes along the weld seam substantially affected the mechanical and macro- and microstructural behavior of the joints. Lammlein et al. [19] employed the FSW process to join small-diameter Al-6061-T6 pipes using a cylindrical threaded pin with a scrolled shoulder. In this investigation, sound joints, in terms of the appearance and tensile strength, were obtained, and the author claimed that the applied geometry could perform FSW with a broad range of process parameters. The application of semiautomatic FSW utilizing a retractable pin to join aluminum 6082-T6 pipes was introduced in Hatting et al.’s [20] work. A 55% weld efficiency was obtained for both the complete tube and micro-tensile samples. Doos et al. [21] demonstrated that the FSW process is feasible for joining Al 6061 pipes. In their work, FS-welded specimens exhibited a joint efficiency of 61.7% by employing the optimum process parameters.

Considering the above mentioned works, only a few studies have been performed on the FSW of metal pipes. Thus, the joining of pipe-shaped samples, particularly polymeric ones, via FSW is a long-standing issue. One of the leading problems that occurs at the curved surface during the connection of the pipeline is the formation of voids on the surface, which was addressed by Mosavvar et al. [22] during FSW of HDPE pipes. However, this work was conducted on small-diameter pipes utilizing a laboratory-scale-type FSW system and, rather than reporting complete tube data, it reported tensile results from specific cut sections, which are affected by the presence of tool pin entrance and exit hole defects. Since weld defects such as residual plunge and extracting holes in the joint are very detrimental to the joint, pipe welding requires a welding tool with a special design. Therefore, the aim of the present work was to design and develop a special rotary FS welding fixture with a retractable pin tool, which can be used for FSW of thermoplastic pipes. The designed orbital FSW could be an efficient and economic technique for high-performance joining of HDPE pipes that has great potential for on-site pipe construction welding compared to other mechanical friction welding methods. As the microstructural and mechanical properties of welded specimens are significantly affected by the FSW process parameters, an investigation of the influence of the tool holder temperature, tool traverse, and rotation speeds was conducted to achieve a higher joint quality and weld strength.

## 2. Materials and Methods

### 2.1. Materials

PE100 HDPE pipes, produced by Sanategharb Company (Tehran, Iran), were used in the present study, since they are widely used for different pipeline applications at different pressures, ranging from drinking water to gas distribution networks, due to their excellent stress cracking, pressure, and impact resistance properties. The HDPE pipe had an outside diameter of 160 mm and a 12.3 mm wall thickness, with an SDR equal to 13. An FSW tool and tool holder were produced with AISI H13 steel material due to its higher thermal conductivity and mechanical properties.

### 2.2. Methods

#### 2.2.1. FSW Tool Development

**Figure 1:**
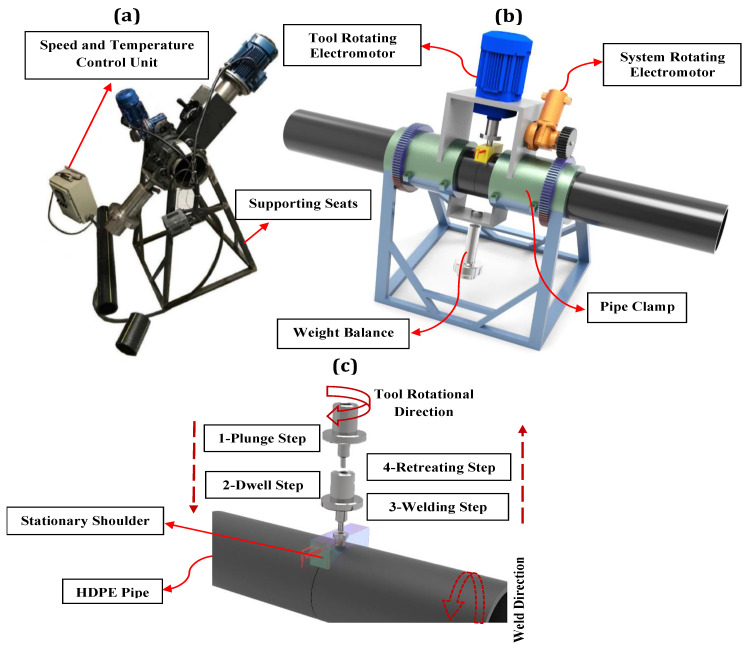
The overall configuration of the orbital FSW system; (**a**) actual picture, (**b**) schematic representation, and (**c**) four steps of the orbital FSW process.

The actual and schematic illustrations of the orbital FSW are depicted in Figure 1. As shown in Figure 1a,b, an outer pipe clamp and supporting seats were designed to stabilize the pipes during the welding process and complete the gear required for rotating the rotary part of the device around the pipes. The rotating part of the device included two electromotors, gearboxes, a tool, and a tool holder mounted on the chassis. A 746-watt electromotor (nominal speed of 1420 rpm) provided the rotational motion of the FSW tool while the tool traverse movement was generated by a 90-watt electromotor (nominal speed of 1350 rpm) that coupled with 2 co-mounted gearboxes with transfer ratios of 1:30 and 1:80. It is worth mentioning that the control of the tool rotational and traverse speeds was possible through two inverters located in the control unit. In the FSW process (see Figure 1c), there are primarily four steps: firstly, the tool plunging step, in which the tool descends to the depth of the workpiece; secondly, the dwell step, during which the tool remains at a constant temperature; thirdly, the welding step, where the joining of the workpiece occurs; and finally, the retreating step where the tool retracts after the welding is completed.

**Figure 2:**
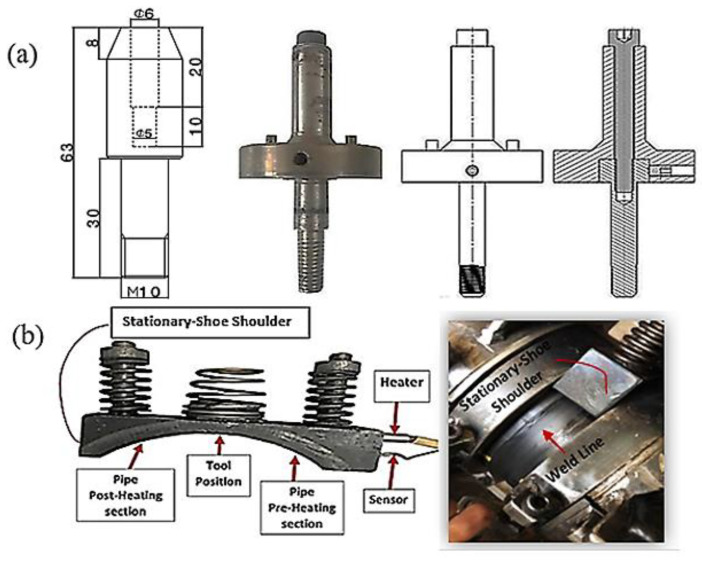
A representation of the FSW tools; (**a**) tool geometry and dimensions and (**b**) stationary-shoe shoulder.

Tool design plays a pivotal role in the FSW of pipes, and a proper design is highly likely to prevent the formation of residual plunge holes at the entrance and exit steps of the tool pin during the welding process. In the presence of a typical rotating FSW tool for the joining of polymeric materials, the formation of external weld defects and undesirable surface quality is a very critical issue. The conventional FSW tool causes outpouring of the soft material from the weld seam, which results in flash defects and imperfect joining. Thus, an FSW tooling system incorporating a stationary shoe shoulder and a retractable tool pin was designed for this purpose. The FSW tool geometry and its dimensions were determined according to the thickness and material of the workpieces as illustrated in Figure 2a. A threaded cylindrical pin profile (M10 × 1) was used for fabricating the joints. Figure 2b shows that to generate a controllable heat during the welding process, a stationary shoe shoulder with an element of 500-watts power was designed and utilized in the tooling system. The controller system carefully monitored the temperature. The surface of the tool holder was coated with polytetrafluoroethylene (Teflon) to prevent the base material from sticking to the stationary shoulder and to achieve a smooth joint seam.

#### 2.2.2. Morphological Analysis of Joints

**Figure 3:**
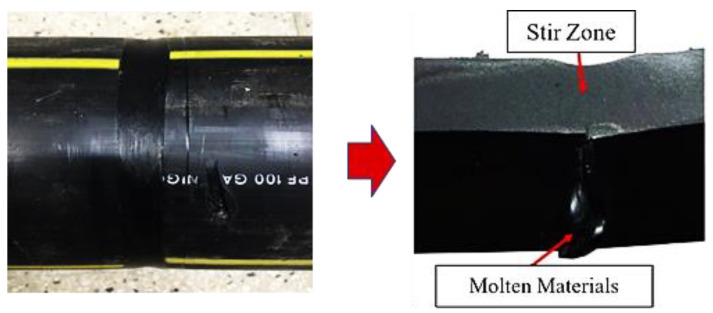
An optimally FS-welded section of HDPE pipe (N1000 V50 T140).

A visual inspection of the welded samples was performed to determine the range of process parameters and curb macroscopic external weld defects. Using the preliminary experimental results, three levels of welding parameters were selected based on the rotational speeds (700, 1000, and 1300 rpm), traverse speeds (25, 50, and 75 mm/min), and shoulder temperatures (120, 140, and 160 °C) as given in Table 1. The cross-sectional view of the optimally welded sample shows the full penetration of the molten materials into the intersection of the two clamped pipes, proving the perfect performance of this device (see Figure 3). As shown in Figure 3, the top view of the welded pipes revealed that the welded samples with a rotation rate of 1000 rpm, transverse speed of 50 mm/min, and stationary shoulder temperature of 140 °C had a uniform appearance compared to the other welded ones. However, the process parameters beyond a specific range provided various external defects, including excessive flash, surface irregularities, and tunnel defects, as described further below.

**Figure 4:**
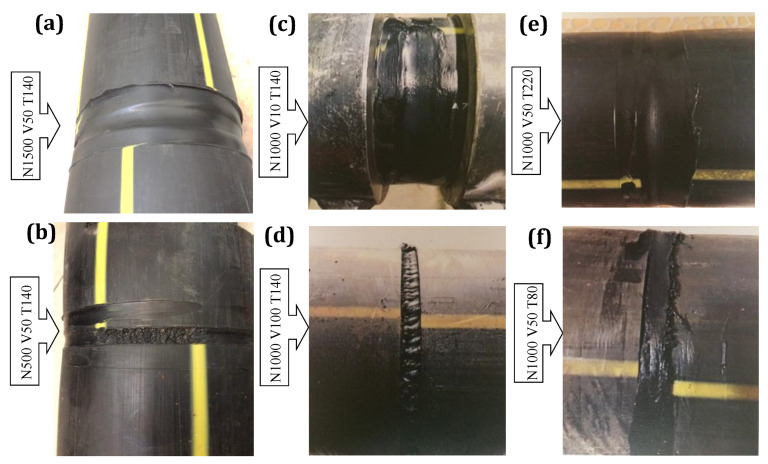
Weld defects formed under an inappropriate set of process parameters (rotational speed, traverse speed and shoulder temperature, respectively): (**a**) 1500 rpm, 50 mm/s, °C; (**b**) 1000 rpm, 10 mm/s; 140 °C; (**c**) 1000 rpm, 50 mm/s, 220 °C; (**d**) 500 rpm, 50 mm/s, 140 °C; (**e**) 1000 rpm, 100 mm/s, 140 °C; (**f**) 1000 rpm, 50 mm/s, 80 °C.

The low rotational speed (500 rpm) produced joints with a porous appearance and discontinuity due to a lack of melting and mixture of the weld materials. Higher rotation rates formed flash defects and reduced the weld thickness, resulting in a low weld strength. FS-welded samples fabricated with the highest (1500 rpm) and the lowest (500 rpm) investigated rotation rates are exhibited in Figure 4a,b, respectively. Other kinds of weld defects, such as surface irregularities and cavities, were generated at the higher traverse speeds while low traverse speeds led to over stirring action and polymer degradation as displayed in Figure 4c,d. Regarding the stationary shoulder temperature, visual inspection revealed that when higher heat generation by an element located in the tool holder during the welding process was used, materials in the weld zone tended to be pushed out, and lateral flashes formed, resulting in a thickness and weld strength reduction (see Figure 4e). However, as illustrated in the macroscopic view of the FS-welded sample in Figure 4f, lower heat generation caused insufficient material mixing to promote the joining in the weld pool.

**Figure 5:**
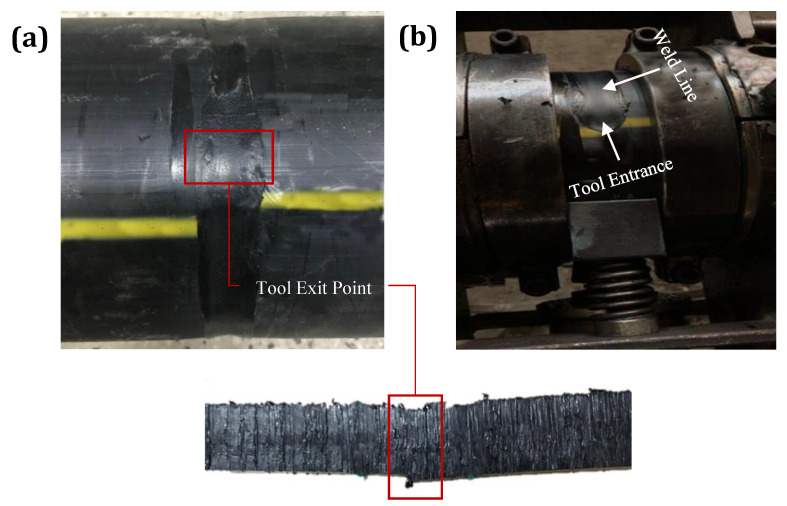
Tool pin’s (**a**) entrance and (**b**) exit points.

During the FSW process, an inappropriate set of welding parameters led to the common weld defects mentioned above. Still, one of the challenging defects that formed at the time of FSW for the pipes was the typical tool exit hole at the end point of the weld line. Inadequate filling of materials in the tool pin exit region was the main reason for this substantial defect in the FSW process. Therefore, the RPT technique was employed to avoid the tool exit hole defect. The top view and cross-sectional view of the weld appearance in the tool pin exit region in Figure 5a shows that gradual tool retraction during weld completion could successfully remove the tool exit hole defect in the FSW of thermoplastic pipes. As insufficient heat generated in the tool pin entrance region could lead to defects, such as material pushing out and the formation of a tool pin entrance hole, this technique was optimized using a stationary shoe shoulder, resulting in a defect-free zone at the weld starting point (see Figure 5b).

### 2.3. Joint Characterization

#### 2.3.1. XRD

Since the crystal morphology of HDPE is highly dependent on the cooling rate, this morphology for both the base material and friction stir-welded sample must be evaluated and compared. To analyze this, the X-ray diffraction method was employed for samples obtained from the stir zone and parent material with dimensions of 10 × 10 × 3 mm^3^. To perform the XRD experiments, an X-ray diffractometer (Bruker-AXS D8-Advance, Karlsruhe, Germany) was utilized using CuKα (λ = 1.541 A) radiation with 2θ set from 5° and 75° at 5°/min.

#### 2.3.2. FTIR

The FT-IR spectra of the base material and stir zone of the FS-welded samples were recorded in the frequency range of 4500–400 cm^−1^ using Fourier transform infrared spectroscopy (Bruker Tensor 27, Bruker, Karlsruhe, Germany). The samples for this measurement were prepared by careful grinding with KBr powder (1:100 ratio) and then the mixtures were pressed into tablets.

#### 2.3.3. SEM

The microstructural characterization and morphology of the fractured surfaces of both the parent material and FSW-welded sample were studied using a scanning electron microscope (SEM) (MIRA3 FEG, Tescan, Brno, Czech Republic). Preceding the SEM observation, specimens were coated with a very thin gold layer and then fractured at a liquid nitrogen temperature.

#### 2.3.4. Mechanical Properties

**Figure 6:**
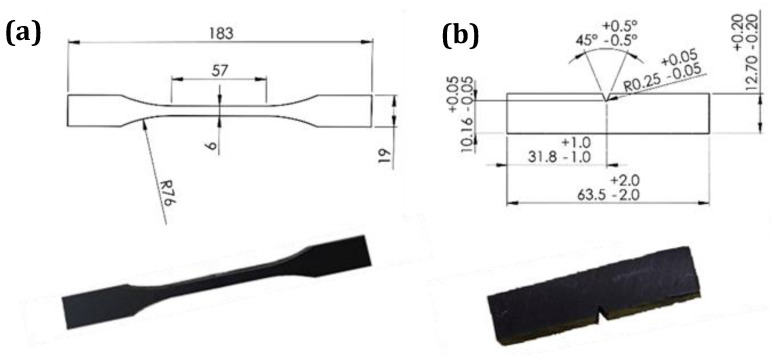
Actual and schematic pictures of (**a**) tensile and (**b**) Izod samples.

The mechanical properties of the base material and FS-welded samples were evaluated using tensile and impact tests. Specimens for the tensile tests were prepared from the pipes in a perpendicular direction to the FSW travelling path with and without the weld. The samples with dimensions based on ASTM D638–02a, type II specification as illustrated in Figure 6a, were cut from straightened plates. The tensile strength of the specimens was measured by a universal testing machine model AI-7000 Taiwan at room temperature and a crosshead speed of 50 mm/ min. The tensile test for each specimen was repeated five times, and the median value with standard deviation was reported.

The Izod impact experiments, on the basis of ASTM D256, were performed on the samples perpendicular to the FSW travelling path with and without the weld, as shown in Figure 6b. All samples were notched using the GT-7016-A2 sampling machine, and V-notches were precisely set on the stir zone of the welded samples. After preparing the specimens, impact tests were conducted by HIT pendulum impact testers (model: HIT25P, Zwick Roell, Ulm, Germany). Five replicates for each sample were performed, and the median value was used.

## 3. Results

Using the orbital friction stir welding system in the present study, defect-free welds for HDPE pipes were obtained within a range of process parameters. According to visual inspection, typical weld defects were successfully removed, and a smooth joint appearance was attained through the FSW tool developments and an appropriate set of parameters. After visual inspection, the mechanical and microstructural characterizations of the FS-welded pipes were analyzed and compared with the base material to understand the prominent attributes of this technique.

### 3.1. Mechanical Properties of Joints

**Figure 7:**
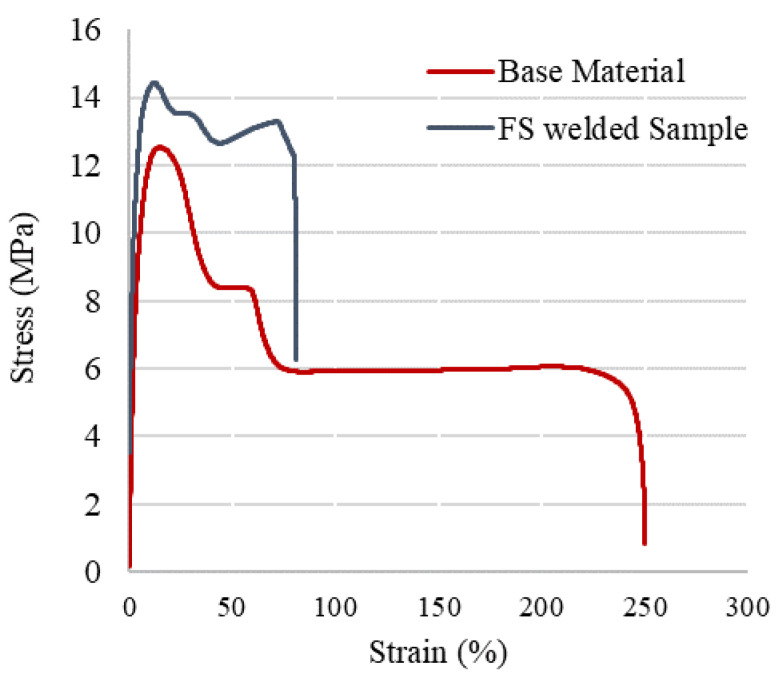
Stress–strain curves of both the base material and the optimally FS-welded samples.

During polymer processing, the process conditions have an influence on the resulting microstructures, which, in turn, determine the properties of the final products. The tensile strength and joint efficiency of the FS-welded samples are the first indicators of the weld quality and mechanical performance of joints. The tensile properties of the HDPE pipes with and without the welding are displayed in Table 2. A statistical analysis using the means of the t-student test was carried out to evaluate whether the differences in the mean values were significant. The 5% percentile was chosen as the level of statistical significance. Since the probability p was lower than 0.05 for all the sample typologies reported in Table 2, the differences between the samples welded with different parameters were considered highly significant. As a result of the macromolecular orientation during extrusion, polymer pipes had a higher axial strength than circumferential strength [23]. Through the FSW process, the circumferential strength of the HDPE pipes increased marginally. In FS-welded HDPE pipes, the increased degree of crystallinity, addition of the molecular orientation, and improvement of the crystalline morphology were responsible for the increased mechanical properties. In addition, the stirring action of the FSW process promoted the orientation of HDPE chains along the hoop direction and thus increased the tensile strength even more than the base material. Therefore, the HDPE pipes with higher performances could be welded by the specific range of FSW parameters. Figure 7 shows the typical stress–strain curve for both the base material and optimally FS-welded samples derived from tensile testing. The welds were orientated perpendicular to the extrusion direction, and the load was applied in the same weld direction for the tensile testing of the welded joints. During the tensile test, welded samples exhibited a brittle fracture, despite the considerable plasticity of the base material samples. A decremental trend can be seen in the strength once the FS-welded joint achieved its maximum strength, indicating that the strength was determined by the ductility of the joints. The strength of the samples welded with the optimized welding conditions was higher than that of the corresponding base material.

**Figure 8:**
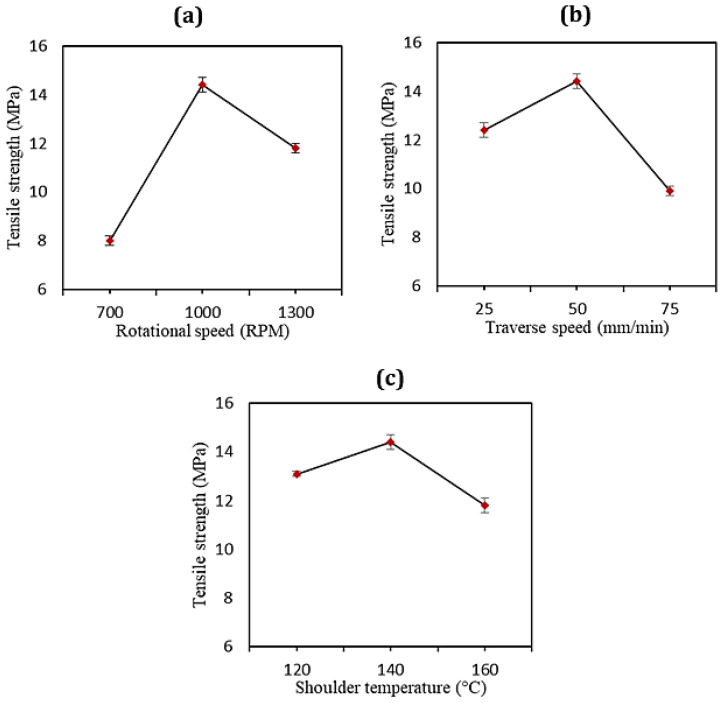
Effects of (**a**) the rotational speed, (**b**) traverse speed, and (**c**) shoulder temperature on the joint strength.

In the FSW of the HDPE pipes, the rotational speed, as the most important factor in comparison with the other process parameters, accounted for the largest impact on the material flow and heat generation as proposed by Bozkurt [12]. The rotational movement of the FSW tool can change the orientation and crystallization behavior of HDPE pipes, leading to the formation of transcrystallinity with a larger crystallite size and to the orientation of the HDPE macromolecular chains in the hoop direction. Thus, the tensile strength of the HDPE pipes joined by the FSW process experienced a slight improvement [24]. In the present case, the main influence of the different tool rotation speeds on the tensile strength of the welded pipes is shown in Figure 8a. According to the obtained tensile results, and compared to the base material, a higher value of the tensile strength (14.4 MPa) was observed at a rotational speed of 1000 rpm. Rotational speeds of 700 and 1300 rpm experienced reductions in the strength of about 45% and 25%, respectively, compared to the base material. This is due to the tool’s rotational velocity being responsible for the production of a larger amount of thermal energy than the other process variables during the FS welding process. The poor tensile strength at lower values of the rotation speed could be attributed to a lack of proper heat generation and strong bond formation. These had detrimental impacts on the mechanical properties of the joints. On the other hand, the high rotation speed during welding caused overflowing and overheating of the HDPE materials, resulting in degradation and burning. In addition, as the rotation speeds increased, a greater amount of stress built up in the hoop directions, which affected HDPE’s crystallization behavior and caused some crystal defects in the bulk materials, deteriorating the mechanical properties of the HDPE pipes.

The effect of the traverse speeds on the tensile strength of the FS-welded HDPE pipes is also displayed in Figure 8b. It shows that for all FSW conditions, by increasing the traverse speed from 25 to 50 mm/min at the same rotational velocity, the tensile strength increased. The low tensile strength at lower traverse speeds can be explained by the longer stirring action time in the weld zone, leading to excessive turbulence in the weld area. When tool traverse speeds of higher than 50 mm/min were used, heat was not adequately generated, and defects formed in the weld pool, thus decreasing the tensile strength.

Figure 8c illustrates the changes in the tensile strength affected by the stationary shoe temperature. Between the shoulder temperatures of 120 and 140 °C, the tensile strength in the welded samples increased from 13.1 MPa to a peak of 14.4 MPa. It then fell to account for the lowest tensile strength at the temperature of 160 °C at around 12 MPa. A proper set of shoulder temperatures and precise control of the thermal energy during the welding process resulted in the increase in the weld strength. In a typical FSW tool, the outer parts of the weld material cool rapidly compared to the inner ones, which causes formation of voids. This defect was addressed through an external heating system in the present study. Therefore, the other advantages of the employed heating system were the lowered cooling rate and the homogeneous distribution of the temperature behind the tool pin along the weld line. Altogether, it was demonstrated that the higher tensile strength for specimens welded under special process conditions (rotation rate of 1000 rpm, traverse speed of 50 mm/min, shoulder temperature of 140 °C) provided excellent outcomes since the tensile strength was comparable to that of the base material and even a slight increase was observed (105%). Similar results are reported in the literature as evidence of the optimum welding conditions, such as, for example, in the case of the ultrasonic welding of E-glass-reinforced polyethylene terephthalate cylinders, where an increase in the shear elastic modulus G’ of 111% compared to the base material was found [6]. The flexural strength of the HDPE plates, as another example, after the optimal FSW process was slightly decreased in comparison with pure HDPE and reached 96% of the base material strength [3].

The dependence of the joint’s impact strength and fracture strain energy on the FSW process was evaluated by comparing the base material and the sample FSW welded under the optimum conditions [25]. The average impact strength value for both the welded sample and base material was 2.45 and 2.74 kJ/m^2^, respectively. Neat HDPE pipes had good toughness while the employed FSW process reduced the toughness of the HDPE pipes. The decrease in the impact strength in the FSW-welded HDPE pipes created regions in which crack initiation required less energy due to the stress concentration. It is worth mentioning that the measured impact strength of the FSW-welded sample was about 89% of that of the base material, indicating the appreciable performance of this method. Since the heat input significantly affected the impact strength, the lower values of the impact strength could be attributed to the insufficient and excessive amount of heat generation during welding, as stated by Aghajani et al. [26]. Thus, monitoring of the heat generation, cooling rate, and entrapping of the weld materials within the weld bead contributed to the higher impact strength in the FSW-welded specimens.

### 3.2. Joints Microstructural Analysis

#### 3.2.1. XRD

**Figure 9:**
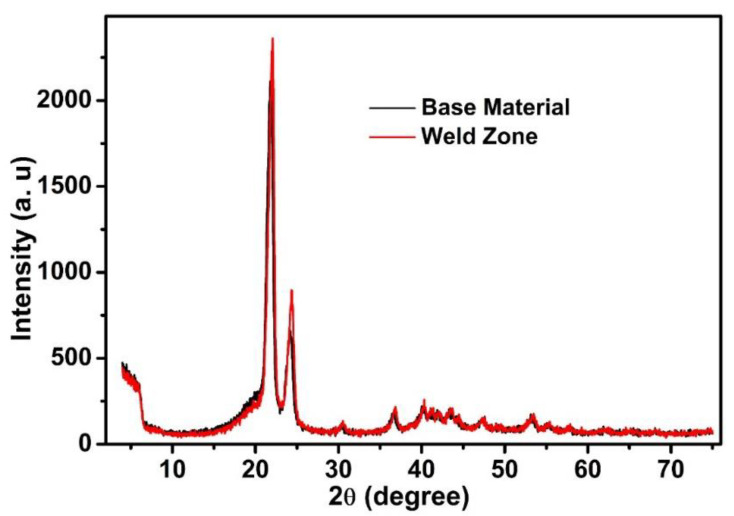
X-ray diffraction patterns attained from pure HDPE and the stir zone.

XRD was performed to evaluate and compare the crystallinity of the optimally FSW-welded HDPE and pure HDPE. Figure 9 exhibits the results of the XRD analysis of the base material and the stir zone. The two major diffraction peaks at about 21.65° and 24.71° in both XRD curves indicated that the FSW-welded sample had similar crystal planes that were consistent with the neat HDPE. An incremental trend was observed in the mechanical properties of the FS-welded sample without alteration of the crystal structure, proving the effectiveness of the proposed orbital FSW system. Using the integrated areas below the crystalline peaks *A_c_* and the broad amorphous halo *A_a_* in the XRD pattern, the degree of crystallinity (*X_XRD_*) was determined from the following equation [27]:

(1) $$X_{XRD=}\frac{A_{c}}{A_{c+}\;A_{a}}$$

Table 3 represents the calculated average crystallinity degree for both the base material and the FSW-welded samples.

It was found that the major peak in the sample after the FS welding was altered to show a sharper form, which was due to the higher crystallinity of the FSW-welded sample than the base HSPE. The decrease in the heat loss and cooling rate at the time of the FSW process contributed to the monitoring of the crystallinity in the welded sample.

The size of the PE crystallites in the base material and FS-welded HDPE samples was calculated from the XRD patterns using the Scherrer equation [28], as given in Equation (2):

(2) $$D\;\left(nm\right)=\frac{K\times \lambda \left(nm\right)}{\beta \left(riadians\right)\times \cos\theta }$$

where *λ* is the wavelength (in nm) of the X-ray radiation used in the diffractometer, *β* is the calculated full width at half height for each crystalline peak (in radians), *K* is a constant related to the crystal structure (0.94 for simple cubic PE crystals), and *θ* is the half of the value of the position of each peak in the 2*θ* axis of the XRD pattern (radians). The values of the main peaks related to each crystal plane were calculated and the average values for the base material and FS-welded zone were obtained. The results indicated that the average crystallite sizes in the base material were about 79 nm, which was reduced to 67 nm in the FS-welded zone of the samples. These crystallites or grains combined to form particles.

#### 3.2.2. FTIR

**Figure 10:**
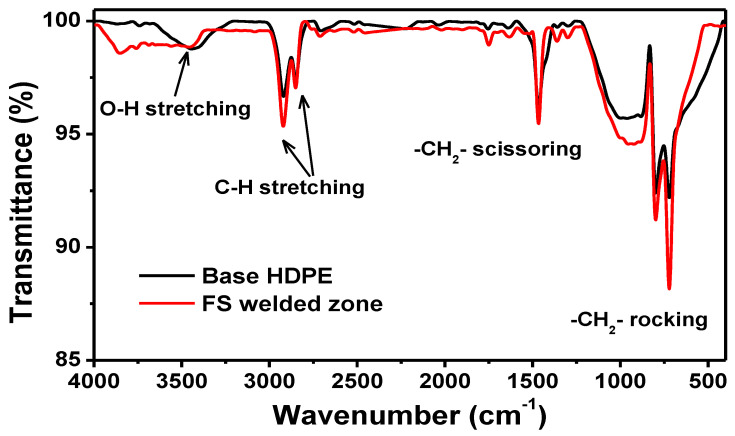
FTIR spectra of the base material and optimally FS-welded HDPE.

The FTIR spectra of the pure HDPE and optimally FSW-welded HDPE are shown in Figure 10. The overall pattern of the spectra for both samples was the same, indicating that there was no difference in the chemical structure of the samples. In the spectra for both samples, the absorption bands appeared in the region of 3443 to 3458 cm^−1^, corresponding to the stretching vibrations of O–H bonds in the moisture absorbed on the PE. The bands at wavenumbers between 2850 and 2920 cm^−1^ represented the C–H stretching vibrations. The absorption band at 1466 cm^−1^ belonged to the in-plane CH_2_ bending vibrations (scissoring mode of –CH_2_). The absorption band at 721 cm^−1^ represented the rocking mode of in-plane bending vibrations of the methylene group (–CH_2_–), which is also known as the long-chain band arising from a chain of more than four –CH_2_– groups bonded together. As mentioned, the generic pattern of the FS-welded HDPE spectra was similar to that of the base HDPE without the appearance of new bands, indicating that no degradation of the existing bonds in the base HDPE or the formation of new bonds occurred during the developed FSW process.

#### 3.2.3. SEM

**Figure 11:**
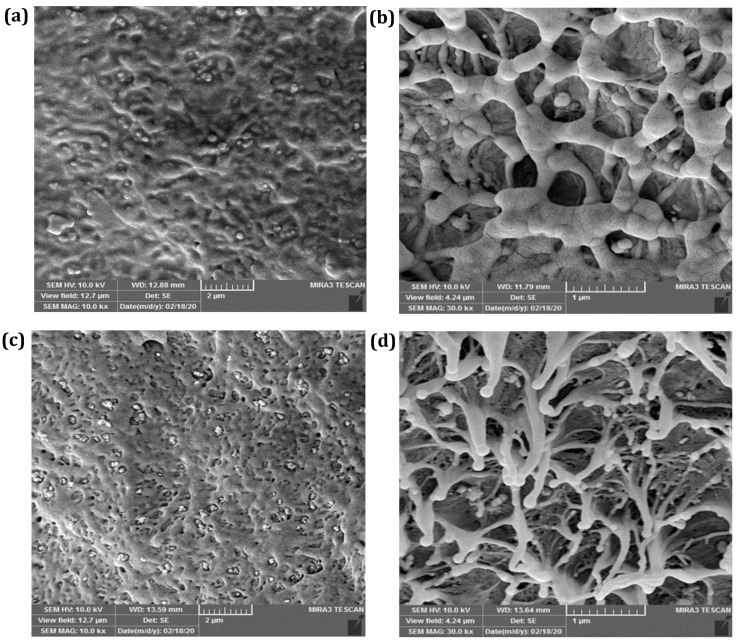
SEM images of (**a**,**b**) base material and (**c**,**d**) optimally FS-welded HDPE pipe (N1000 V50 T140) under different magnifications.

The microstructure of the pure HDPE and the stir zone fabricated under optimum conditions is shown in Figure 11. After the FSW process, the amount of fine crystals in the HDPE samples was slightly increased, resulting in a higher degree of crystallinity of the FS-welded zone in comparison to the base HDPE. These observations agreed with the XRD results, which showed an increment in the crystallinity of the welded zone. The materials in both the pure HDPE and stir zone possessed a lamellar structure; however, the lamellar size of HDPE was reduced after the welding procedure. Using image processing software and measurement of the size of crystallites in the SEM images, it was determined that the average size of the crystallites dropped from 360 nm in the base HDPE samples to 290 nm in the FS-welded zone (Figure 11). These results showed the same trend as the changes observed in the XRD patterns of the size of the crystallites.

The degree of crystallinity in the developed mechanism of the FSW process was more controllable compared to the fusion welding methods. Based on this, monitoring of the temperature during FS welding and appropriate selection of the welding conditions can significantly contribute to achieving the desirable mechanical and microstructural properties of FS-welded HDPE pipes.

## 4. Conclusions

A heating-assisted approach to FSW was developed and implemented for HDPE pipes with an outside diameter of 160 mm and a wall thickness of 12.3 mm. The concluding remarks could be drawn as follows:

- The joining of HDPE pipes with desirable combinations of tensile and impact properties was successfully performed using the specially designed, portable, and heating-assisted FSW system.
- The proposed technique, utilizing a stationary shoe shoulder with a retractable tool pin, was able to fabricate sound joints without the appearance of tool exit hole defects.
- Microstructural observations of the stir-welded zone using XRD, FTIR, and SEM techniques revealed that the crystal structures of the FS-welded sample were similar to the base material, proving the reliability of the developed system.
- The samples welded with the optimum parameters presented comparable properties to the base materials and even a slight increase in the tensile strength (105%).
- The maximum impact strength was 2.45 kJ/m^2^, which represented about 89% of the base HDPE impact strength.

## Figures and Tables

**Table 1:** FSW parameters, limits, and levels.

Process Parameters	Rotational Speed (rpm)			Traverse Speed (mm/min)			Shoulder Temperature (°C)			Tool Tilt Angle
Unit level	L1	L2	L3	L1	L2	L3	L1	L2	L3	constant
Unit value	700	1000	1300	25	50	75	120	140	160	0°

**Table 2:** Process parameters and corresponding tensile test results.

Sample	Tool Rotation Rate (rpm)	Tool Traverse Speed (mm/min)	Shoulder Temperature (°C)	Tensile Strength (MPa)	Joint Efficiency (%)
Base Material	-	-	-	13.6	100
1	700	50	140	8 ± 0.2	59
2	1000	25	140	12.4 ± 0.3	91
3	1000	50	140	14.4 ± 0.2	105
4	1000	75	140	9.9 ± 0.2	73
5	1000	50	120	13.1 ± 0.1	96
6	1000	50	160	11.8 ± 0.3	87
7	1300	50	140	10.8 ± 0.2	80

**Table 3:** Crystallinity degree calculated from XRD measurements.

Sample	*X_XRD_* (-)
Pure HDPE	0.703
FS-welded	0.734

## Data Availability

Data is contained within the article.

## Nomenclature

**OFSW**	Orbital friction stir welding
**FSSW**	Friction stir spot welding
**HDPE**	High density poly ethylene
**PEEK**	Poly-ether-ether-ketone
**NBR**	acrylonitrile-butadiene rubber
**XRD**	X-ray diffraction
**FTIR**	Fourier transform infrared spectroscopy
**MWCNTs**	Multiwalled carbon nanotubes
**PA6**	Polyamide6
**ABS**	Acrylonitrile butadiene styrene
**HNTs**	Halloysite nanotubes
**RPT**	Retractable Pin Tool
**SDR**	Section diameter ratio
**SEM**	Scanning electron microscope
